# Vaccines Using *Clostridium perfringens* Sporulation Proteins Reduce Necrotic Enteritis in Chickens

**DOI:** 10.3390/microorganisms10061110

**Published:** 2022-05-27

**Authors:** Ying Fu, Mohit Bansal, Tahrir Alenezi, Ayidh Almansour, Hong Wang, Xiaolun Sun

**Affiliations:** 1 Center of Excellence for Poultry Science, University of Arkansas, Fayetteville, AR 72701, USA; yingfu@uark.edu (Y.F.); mb043@uark.edu (M.B.); tjalenez@uark.edu (T.A.); ama056@uark.edu (A.A.); hxw01@uark.edu (H.W.); 2 Cell and Molecular Biology (CEMB), University of Arkansas, Fayetteville, AR 72701, USA

**Keywords:** sporulation, inflammation, growth performance, histopathology

## Abstract

*Clostridium perfringens* is the prevalent enteric pathogen in humans and animals including chickens, and it remains largely elusive on the mechanism of *C. perfringens*-induced enteritis because of limited animal models available. In this study, we investigated the role of *C. perfringens* sporulation proteins as vaccine candidates in chickens to reduce necrotic enteritis (NE). *C. perfringens* soluble proteins of vegetative cells (CP-super1 and CP-super2) and spores (CP-spor-super1 and CP-spor-super2) were prepared, and cell and chicken experiments were conducted. We found that deoxycholic acid reduced *C. perfringens* invasion and sporulation using the *Eimeria maxima* and *C. perfringens* co-infection necrotic enteritis (NE) model. *C. perfringens* enterotoxin (CPE) was detected in the CP-spor-super1&2. CP-spor-super1 or 2 induced cell death in mouse epithelial CMT-93 and macrophage Raw 264.7 cells. CP-spor-super1 or 2 also induced inflammatory gene expression and necrosis in the Raw cells. Birds immunized with CP-spor-super1 or 2 were resistant to *C. perfringens*-induced severe clinical NE on histopathology and body weight gain loss. CP-spor-super1 vaccine reduced NE-induced proinflammatory *Ifnγ* gene expression as well as *C. perfringens* luminal colonization and tissue invasion in the small intestine. Together, this study showed that CP-spor-super vaccines reduced NE histopathology and productivity loss.

## 1. Introduction

*Clostridium perfringens*, a Gram-positive and spore-forming anaerobe, has been responsible for intestinal diseases in animals and humans over centuries [1]. The bacterial pathogen is one of the top five foodborne pathogens [2] and causes more than one million cases of foodborne and nonfoodborne enteritis yearly in the United States [3]. *C. perfringens* causes human enteritis necroticans, acute watery diarrhea (food poisoning), non-foodborne diarrhea, preterm infant necrotizing enterocolitis (NEC) and Darmbrand, as well as animal enteritis including equine acute NEC and swine enterocolitis [1]. *C. perfringens* also induces necrotic enteritis (NE) in chickens often predisposed to coccidia *Eimeria Maxima* or *Eimeria acervuline* [4], eating a high fishmeal [5] or high fiber diet [6]. NE has reemerged as one of the prevalent diseases in the poultry industry because of trending restriction of prophylactic antibiotic usage in food animal production [7]. NE costs around $6 billion every year worldwide [8]. Clinical NE causes up to 50% mortality [9], while subclinical NE is more common without peak mortality.

Despite the different clinical signs of *C. perfringens*-induced enteritis among animals, one of the most shared features is the histopathology manifestations with the inflammatory necrotic destruction of intestinal villi and/or crypts in humans and animals [10,11,12,13]. The histopathology lesion is mainly resulted from combined events of bacterial overgrowth [14,15], gas accumulation [16], collateral inflammatory self-destruction [10], and toxin production [1]. Different strains of *C. perfringens* produce a variety of toxins and enzymes including alpha (CPA), beta (CPB), epsilon (ETX), iota (ITX), enterotoxin (CPE), necrotic enteritis B-like toxin (NetB), and others [17]. NetB but not CPA has been implicated in chicken NE [18]. Although NetB-positive *C. perfringens* induce NE in certain experimental settings [19], the development of NE is not always associated with the presence of NetB, CPB2, or TpeL [20,21]. NetB-positive *C. perfringens* alone did not always induce NE or chicken body weight gain loss [22,23,24]. The presence of a high dose of *C. perfringens* in the intestine does not sufficiently induce NE, and its counts alone do not associate with NE [25,26,27].

*C. perfringens* enterotoxin (CPE) is responsible for human food-poisoning and non-foodborne *C. perfringens*-mediated enteritis/diarrhea [28]. CPE is produced and released only when *C. perfringens* sporulates–but not in vegetative growth–in the intestine in response to stressed conditions. Intestinal epithelial cell (IEC) lines [29], ex vivo tissue [30], and the intestine loop challenge model [31,32] have been used to reveal the mechanism of CPE on epithelial cell death. CPE binds to IEC tight junction protein claudins to form membrane pores and to cause cell death [29,32]. Although these findings have greatly advanced our understanding of CPE on IEC death, a number of knowledge gaps on sporulation, CPE, and enteritis remain elusive, such as the role of immune cells, inflammatory signaling, and microbiota. The limitation in understanding, in part, comes from few available animal models for longer (>12 h) and “natural” (non-surgery) *C. perfringens* infection in the presence of CPE. Interestingly, *C. perfringens*-induced NE lasts more than two days and has been studied in chickens for decades. NE birds show watery diarrhea and inflammation in the small intestine [10], resembling human *C. perfringens*-induced enteritis [30]. It is well-known that predisposing factors such as coccidiosis and a high fishmeal diet are important for initiating *C. perfringens*-induced NE [33]. These factors cause inflammation and/or dysbiosis and may facilitate *C. perfringens* overgrowth and induction of the enteritis. Accordingly, various infection models have been developed to recapitulate NE pathogenesis, while NE in commercial poultry production is often associated with coccidiosis and more complex enteritis compared to the *C. perfringens* infection alone model. Together, the mechanism of *C. perfringens*-mediated enteritis including NE remains largely inconclusive.

The NE model in our lab is based on *Eimeria* and *C. perfringens* co-infection to mimic commercial poultry production settings. We previously found that secondary bile acid deoxycholic acid (DCA) [10,34] and lithocholic acid [14] prevented clinical and subclinical NE and reduced *C. perfringens* colonization, invasion, and sporulation. We reasoned that the success of DCA against NE could come from reducing *C. perfringens* sporulation and associated pathogenicity. In this study, we aimed to address this hypothesis using molecular studies as well as chicken immunization and challenge experiments. This study showed the vaccines using *C. perfringens* sporulation proteins prevented the severe clinical NE development. The findings will be important for designing new strategies against *C. perfringens*-induced enteritis in humans and animals.

## 2. Materials and Methods

### 2.1. Extraction of Soluble Proteins from C. perfringens Sporulation and Vegetative Cultures

*C. perfringens* isolate of CP_2 has been used in previous reports in our lab [10,14,34] and was sourced from USDA-ARS, College Park, TX, USA [6,35]. *C. perfringens* isolate of CP1 has been recently isolated from NE birds in our lab and induced more severe NE in chickens compared to CP_2 (data not shown). CP1 and CP_2 were vegetatively grown in Fluid Thioglycollate (FTG, BD Bioscience, Franklin Lakes, NJ, USA) broth or induced sporulation in Duncan Strong Sporulation Medium (DSSM, HiMedia Laboratory, West Chester, PA, USA). Soluble proteins from the FTG non-sporulation or DSSM sporulation supernatant of CP1 and CP_2 were individually extracted following the protocol [36,37] without column purification. Briefly, *C. perfringens* in stock meat broth was inoculated into fresh FTG broth and heated at 72 °C for 20 min to kill vegetative cells, followed by culturing anaerobically at 42 °C for 18 h. The bacterium was then inoculated into large scale fresh FTG broth for an additional 9 h culture. Next, the bacterium was inoculated to DSSM and cultured for 8 h at 42 °C. The medium was centrifuged twice at 5000 rpm for 20 min at 4 °C and the supernatant was collected. An equal amount of 80% saturated ammonium sulfate was gradually added to the supernatant. The supernatant was then centrifuged at 10,000 g for 20 min at 4 °C. The resulted pellet was dissolved in 1× PBS (pH 6.8) and labelled as CP-spor-super 1 or 2. To collect the supernatant protein of CP1 and CP_2 grown vegetatively, the bacteria were cultured in FTG broth overnight. The soluble protein in the supernatant was isolated using ammonium sulfate method as described above and named CP-super1 and CP-super2. The protein concentrations in CP-super1, CP-super2, CP-spor-super1, and CP-spor-super2 were measured by Bio-Rad assay as 1.2, 2.1, 1.4, and 4.3 mg/mL, respectively. All the used supernatants were prepared once.

### 2.2. C. perfringens Toxinotyping

CP1 or CP_2 was grown in Tryptic Soy Broth (TSB) overnight, and DNA was extracted from the cell pellet using sonication, boiling, and QIAquick PCR purification. Briefly, the cell pellet resuspended in 300 µL PBS and 20 µL 10% SDS was sonicated at 70% duty cycles, output control at 6 for 15 s on a Branson Sonifier 450 (VWR, Radnor, PA, USA). After centrifugation, the supernatant was boiled at 100 °C for 20 min. After centrifugation again, DNA in the supernatant was purified with QIAquick PCR kit (Qiagen, Hilden, Germany). The bacterial strains were toxinotyped with Taq PCR kit (Neb, Ipswich, MA, USA) and the PCR products were visualized on 2% agarose gel using the Odyssey Fc Imaging System. The PCR program was 95 °C for 2 min, then 35 cycles of 95 °C for 20 s, 56 °C for 20 s, 68 °C for 45 s. *C. perfringens* genomic and plasmid DNA sequences of *C. perfringens* (NC_003366.1, NC_015712.1, NC_007773.1, NC_011412.1, NC_008261.1, and NC_019688.1 at NCBI) were obtained to design primer sequences with Primer 3 [38,39] and the sequences of these primers were listed in Supplementary Table S1.

### 2.3. Challenge Mouse Intestinal Epithelial Cell and Macrophage with the Four Supernatants

Mouse intestinal epithelial CMT-93 cells (ATCC, Manassas, VA, USA) were cultured in 10% FBS high glucose DMEM medium (ThermoFisher, St. Louis, MO, USA). Raw 264.7 macrophage (ATCC, Manassas, VA, USA) was cultured in 10% FBS low glucose DMEM medium (ThermoFisher, St. Louis, MO, USA). To dissect the effect of the CP1 and CP_2 supernatants on intestinal tissue and cell response, the cells were plated at 2 × 10^4^ cells/well of 8-well cell culture chamber slide and challenged with CP-super1 (8.5 µL/mL), CP-super2 (4.9 µL/mL), CP-spor-super1 (7.1 µL/mL), or CP-spor-super2 (2.4 µL/mL) for 24 h. Propidium iodide (PI) staining was used to show the dead cells in red fluorescence. To examine the inflammatory response induced by CP1 and CP_2 supernatants, the Raw cells at 2 × 10^6^ cells/well of 6-well plate were challenged with the same concentration of CP-super1, CP-super2, CP-spor-super1, or CP-spor-super2 for 4 h, and the cells were lysed in Trizol for RNA isolation. To further assess which cell death pathway(s) were induced by the supernatants, the Raw cells in 6-well plate were challenged with the four supernatants or murine inflammatory cytokine TNFα (5 ng/mL) as control for 5 h, and the cells were lysed in 1x Laemmli buffer. The protein concentration was finally quantified with Bio-Rad assay.

### 2.4. Western Blotting

The protein samples at 20 μg/lane were separated on a 10% SDS-PAGE gel, transferred onto a nitrocellulose membrane, and blocked in 5% BSA TBST buffer. The membrane was then incubated with 1:1000 dilution of primary rabbit polyclonal anti-*C. perfringens* enterotoxin (CPE) (cat# 64-052 BioAcademia, Suita, Osaka, Japan), anti-p-MLKL (cat# 37333), anti-cleaved Caspase 3 (cat# 9664), anti-RIP3 (cat# 15828), or anti-Actin (cat# 4970S) antibody (Ab) (the latter four rabbit anti-mouse mAb from Cell Signaling, Danvers, MA, USA) overnight at 4 °C. After washing, the membrane was incubated with secondary Ab of 1:1000 diluted HRP conjugated donkey anti-rabbit IgG (cat# A16035 H+L, ThermoFisher, St Louis, MO, USA). The Western Blot results were imaged using an Odyssey Fc Imaging System (LI-COR Biosciences, Lincoln, NE, USA). The density of Western blot bands was quantified using the ImageJ [40] Gel Analysis method, and data were normalized to Actin control as described before [41].

For the Western Blot of chicken sera, group-pooled sera from birds of noninfected (NC), NE, or NE, CP1-1, CP1-2, and CP2 was used as primary Ab. 20 μg of CP-spor-super1 per lane was separated on a 10% SDS-PAGE gel, transferred onto a nitrocellulose membrane, and blocked in 5% BSA TBST buffer. The membrane was separated into individual pieces and incubated with 1:300 diluted group-pooled chicken sera as primary Ab. After washing, the membrane was blotted with secondary Ab of 1:1000 diluted HRP Conjugated goat anti-chicken IgY (H & L) (cat# PA1-28798, ThermoFisher, St. Louis, MO, USA). A separate membrane was also blotted with anti-CPE primary and anti-rabbit secondary Ab. The individual membranes were then imaged and aligned together based on the protein molecular weight marker.

### 2.5. Total RNA and DNA Isolation

For RNA isolation, at the end time points of Raw 264.7 cell experiment, the cells were lysed with 1 mL Trizol (ThermoFisher, St. Louis, MO, USA) and stored at −80 °C before RNA isolation. Chicken intestinal tissue (around 0.1 g) stored at −80 °C was added with 1 mL Trizol and 200 μL 0.1 mm zirconia beads (BioSpec Products, Bartlesville, OK, USA) and was immediately bead-beaten at 5 m/s for 3 × 30 s on a Fisherbrand™ Bead Mill 24 Homogenizer (ThermoFisher, St. Louis, MO, USA). The samples were immediately cooled on ice and stored at −80 °C before RNA isolation. RNA was isolated as reported before [42]; briefly, the stored samples suspended in Trizol were thawed, and 200 µL of chloroform was added per 1 mL of Trizol Reagent. The mixture was vortexed thoroughly for 30 s, sat at room temperature for 10 min, and centrifuged at 12,500 rpm for 14 min at 4 °C. The top 500 μL colorless aqueous phase was then transferred to a fresh 1.5 mL microcentrifuge tube. The RNA was precipitated by mixing the aqueous phase with 500 μL isopropyl alcohol and incubating at room temperature for 10 min. After centrifugation, the pellet was washed once with 1 mL 75% ethanol and centrifuged again. The dried pellet of RNA was then dissolved in RNase/DNase free water. RNA was quantified with NanoDrop (Thermo Fisher Scientific, Waltham, MA, USA) and the RNA purity was evaluated based on ratios of 260/280 and 260/230. The RNA was reverse-transcribed into cDNA using M-MLV (NE Biolab, Ipswich, MA, USA) and random hexamer.

DNA was extracted from small intestinal content and tissue of d 21 birds using the phenol/chloroform method as previously described [43]. Briefly, content and tissue samples at the small intestine were weighted and transferred to 2 mL screw cap tubes containing 200 µL 0.1 mm zirconium beads and added with 500 μL PBS, 85 μL 10% SDS solution and 500 μL Phenol: Chloroform (25:24) (using the lower part in the mixture). The samples were bead-beaten at 5.5 m/s for 3 × 30 s. DNA in the top 500 μL layer was extracted one more time with 500 μL Phenol: Chloroform and once with 500 μL chloroform. The top 500 μL DNA layer was precipitated by mixing with 50 μL 3M sodium acetate (pH 5.2) and 1.25 mL 100% ethanol and stored at −20 °C overnight. After centrifugation, the DNA pellet was washed once with 70% ethanol. The dried DNA pellet was dissolved in RNase/DNase free water. The DNA was diluted 10 or 100 times before real time PCR assay to reduce the interference from residue DNA polymerase inhibitors in the intestinal content.

### 2.6. Real Time RT-PCR

mRNA levels of proinflammatory genes of mouse *Il1β*, *Tnfα*, and *Cxcl2*, and chicken *Ifnγ*, *Il1β*, and *Il8-1*, were determined using the SYBR Green PCR Master mix (Bio-Rad Hercules, CA, USA) on a Bio-Rad 384-well Real-Time PCR System and normalized to the respective *Gapdh*. The primer sequences were reported before [10,14,42] and are in Supplementary Table S1. The PCR reactions were performed according to the manufacturer’s recommendation. Similarly, the level of *C. perfringens* in the intestinal luminal and tissue was also quantified by RT-PCR targeting the bacterial 16S rDNA, as described before [14].

### 2.7. Chicken Experiment

The chicken experiments were conducted at the Poultry Health Laboratory of University of Arkansas at Fayetteville. Animal experiments performed were in accordance with the Animal Research: Reporting of In Vivo Experiments (https://www.nc3rs.org.uk/arrive-guidelines (accessed on 18 December 2021)). All the experimental procedures were approved by the Institute Animal Care and Use Committee of the University of Arkansas. Zero-day-old broiler chicks were obtained from Cobb-Vantress (Siloam Springs, AR, USA). Upon arrival, broiler chicks were neck-tagged, individually weighed, and randomly assigned to cage pens in a controlled age-appropriate environment. Birds were fed with basal diets from d 0 to d 21. The basal diets were a corn-soybean meal-based starter diet during d 0 to 10 and a grower diet during d 11 to 21 [10]. Birds were immunized with subcutaneous injection of CP-spor-super1 and 2 vaccines with Aluminum Hydroxide Gel Adjuvants (ThermoFisher, St Louis, MO, USA) at d 0. CP-spor-super1 vaccine was used with two levels of doses at 0.141 (CP1-1, 1000× dilution of the CP1 supernatant soluble proteins) and 0.704 µg/bird (CP1-2, 200× dilution of the CP1 supernatant soluble proteins), while CP-spor-super2 vaccine was used at 0.043 µg/bird (CP2, 10,000× dilution of the CP_2 supernatant soluble proteins). In the previous vaccine dose titration experiments (data not shown), CP-spor-super1 showed better protection against NE compared to CP-spor-super2. We then used the two-dose groups of CP-spor-super1 and one-dose group of CP-spor-super2. A second booster immunization (10× more than first doses) was conducted at d 10. The birds at d 0 were used as following: non-infected (NC) group: 13 birds; NE group: 16 birds; CP1-1 group: 16 birds; CP1-2 group: 7 birds; and CP2 group: 7 birds. Bird numbers at d 21 were NC: 12; NE: 14; CP1-1: 16; CP1-2:7; and CP2:7. Birds were infected with 20,000 sporulated *E. maxima* M6 oocysts at d 16 similar to described before [14]. The birds were then infected with *C. perfringens* CP1 at 10^9^ CFU/bird at d 20 similar to described before [10]. Chicken body weight was measured at d 0, 16, 20, and 21. Birds were euthanized at d 21 to collect blood and small intestinal tissue and content for analysis of antibody titer, histopathology, inflammation, and *C. perfringens* colonization and invasion levels.

### 2.8. ELISA

Sera collected from groups of NC, NE and CP1-2 was used for the evaluation of Ab titer using ELISA assay. Briefly, a 96-well ELISA plate was coated with triplicated 50 μL/well of diluted antigen (CP-spor-super1) at a final concentration of 3.0 μg/mL and incubated at 4 °C overnight. The plate was washed three times with 200 μL TBST buffer, followed by adding 200 μL blocking buffer (5% BSA in TBS) at 37 °C for 2 h. 100 μL 1:100 diluted chicken sera in blocking buffer was added into the wells as the primary Ab and incubated at 4 °C overnight. After being washed three times with 200 μL TBST buffer, 100 μL 1:10,000 diluted HRP Conjugated Goat anti-Chicken IgY (H & L) was added as secondary Ab and incubated for 1 h at 37 °C. For signal detection, 100 μL TMB substrate mixture was added. The color development was stopped 15 min later by adding 100 μL of 2 M H_2_SO_4_ per well. Optical density at 450 nm (OD_450_) was measured by BioTek 800TS Microplate Absorbance Spectrophotometer (Agilent, Santa Clara, CA, USA).

### 2.9. Histopathology Analysis of Intestinal Inflammation

At d 21, tissue of the jejunum (2 cm) and ileum (8 cm) adjacent to Meckel’s diverticulum was resected, Swiss-rolled, and fixed in 10% phosphate-buffed formalin (pH 7.4) overnight at 4 °C, as describe before [10]. Tissue samples were then embedded in paraffin and 5 μm thick sections were cut, processed, and stained with H & E at the Histology Laboratory at Department of Poultry Science at University of Arkansas at Fayetteville. The H & E slides were evaluated for intestinal lesions basing on the level of immune cell infiltration in the lamina propria, villus length, crypt hyperplasia, and ulceration as described before [10]. Images of representative histopathology were acquired using a Nikon TS2 fluorescent microscope.

### 2.10. Statistical Analysis

Differences between treatments were analyzed using One-way ANOVA followed by Bonferroni pairwise comparison using Prism 7.0 software. Data were also analyzed using the nonparametric Mann–Whitney *U* test. The specific pairwise comparisons were showed in Results section and Figures. Values are shown as the mean of samples in the treatment ± standard error of the mean as indicated. Experiments were considered statistically significant if *p* values were <0.05.

## 3. Results

### 3.1. Dietary DCA Reduced C. perfringens Invasion and Sporulation

Based on previous studies on DCA against *C. perfringens* sporulation [34], we used modified FISH assay with the pathogen 16S rDNA FISH probe to examine additional histology slides from NE and DCA-treated birds in a previous trial [10]. From the FISH images, numerous *C. perfringens* were shown to invade and sporulate (ball-shaped red spores, pointed yellow arrows) deep inside intestinal villi and lamina propria of the NE birds (Supplemental Figure S1, left panels). Notably, DCA reduced *C. perfringens* invasion and sporulation, showing a few red rod-shaped vegetative cells (pointed green arrow, right panels). Based on the result that dietary DCA reduced severe NE in chickens [10] and DCA reduced *C. perfringens* sporulation in the small intestine of NE birds, it was supported that DCA-reduced NE was related to *C. perfringens* sporulation.

### 3.2. Soluble Proteins from C. perfringens Sporulation Supernatant Induced Cell Death in Intestinal Epithelial Cell and Macrophage

**Figure 1:**
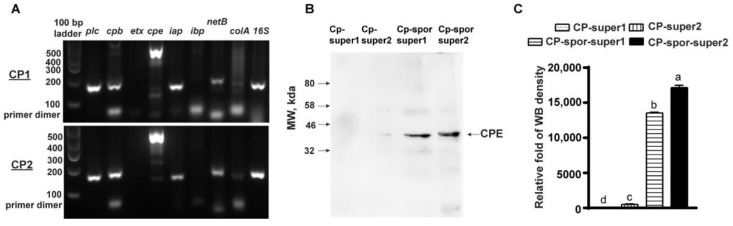
*C. perfringens toxinotype* and CPE expressed in CP1 and CP_2. (**A**). CP1 and CP_2 DNA was toxinotyped by PCR. Showed were the PCR gel image. (**B**). CP1 and CP_2 were vegetatively grown in FTG or induced sporulation in DSSM, and the soluble proteins were isolated by precipitating using ammonium sulfate method. After dissolved in pH 6.8 PBS, the soluble supernatant proteins were subjected to Western Blot analysis with anti-CPE primary Ab. (**C**). WB quantification using ImageJ. The Western Blot image of CPE expression is shown. Different letters mean significant.

To investigate whether *C. perfringens* sporulation influenced NE outcome, *C. perfringens* single colony isolates of CP1 and CP_2 were used. CP_2 was used in the previous studies [10,14,34] and CP1 was newly isolated at the farm. The CP1 and CP_2 were toxinotyped using PCR and gel imaging. Both CP1 and CP_2 were genomic/plasmid DNA positive for *plc*, *cpb*, *cpe*, *iap*, *netB*, and *colA*, while both of them were negative for *etx* and *ibp* (Figure 1A). Because the toxin genes except *cpe* were evaluated at the DNA level without RNA or protein level evaluation, the results do not exclude the possibility of silence toxin genes or non-functional toxin proteins. At the DNA level, both isolates did not fall into any current toxinotype category [44], although they were derived from individual single colonies.

CP1 and CP_2 were vegetatively grown in FTG or induced sporulation in DSSM. Soluble proteins from *C. perfringens* vegetative or sporulation supernatant were extracted following the protocol [37] and labeled as CP-super1, CP-super2, CP-spor-super1, and CP-spor-super2 without column purification. The presence of CPE in these four supernatants was evaluated using Western Blot assay with anti-CPE antibody [45]. As showed in Figure 1B,C, the level of CPE was higher in CP-spor-super2 compared to CP-spor-super1 or vegetative growth supernatants. The results suggest that both isolates were positive for the sporulation and CPE production.

**Figure 2:**
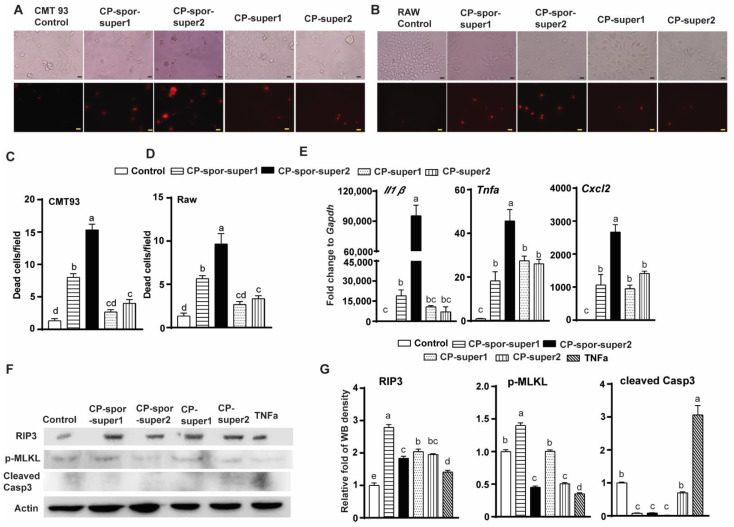
CP-spor-super1 or 2 induced intestinal epithelial cell (IEC) and macrophage cell necrosis. Cells were challenged with CP-super1&2 and CP-spor-super1&2. (**A**,**C**) Mouse IEC CMT-93 for 24 h (red of dead cells, propidium iodide (PI) staining). (**B**,**D**) Mouse Raw 264.7 macrophage for 24 h. (**E**) Raw cells to assess mRNA expression for 4 h. (**F**) Raw cell to assess protein expression by WB for 5 h. (**G**) WB quantification using ImageJ. All graphs depict mean ± SEM. Different letters mean significant. Results are representative of three independent experiments. Scale bar is 20 μm.

To functionally dissect the role of the CP-spor-super on cell activities, mouse intestinal epithelial cell CMT-93 and murine macrophage Raw 264.7 cells were challenged with soluble supernatant proteins of CP-spor-super1 or 2 from sporulated bacteria or CP-super1 or 2 from vegetative bacteria. After 24 h incubation, more cell death (red, propidium iodide (PI) staining) was observed in the CMT-93 cells challenged with CP-spor-super1 or 2 compared to CP-super1 or 2 (Figure 2A,C). Consistently, death was induced in the Raw cells when challenged with the sporulation proteins for 24 h (Figure 2B,D). These results suggest that *C. perfringens* sporulation produced proteins toxic to cells.

Cell death could be a direct result of toxin action and/or an indirect event through induction of inflammation. We then examined whether CP-spor-super1 or 2 induced the inflammatory response using the Raw cells. The macrophage was challenged with the four supernatants for 4 h, and proinflammatory gene expression was assessed by real time PCR. Notably, all four *C. perfringens* supernatants induced strong proinflammatory mRNA *Il1β*, *Tnfα*, and *Cxcl2*, while CP-spor-super2 induced more inflammatory cytokine compared to other supernatants (Figure 2E). It is well documented that an overzealous inflammatory response often induces cell death. To further assess which cell death pathway(s) were activated by the supernatant proteins, the Raw cells were challenged with the four supernatant proteins or murine inflammatory cytokine TNFα for 5 h. Unlike inflammatory cytokine TNFα, CP-spor-super1 increased expression of necroptosis marker proteins RIP3 and p-MLKL but not cleaved Caspase 3 of apoptosis marker (Figure 2F,G), suggesting CP-spor-super1 inducing necrosis but not apoptosis in the Raw cells. Interestingly, CP-spor-super2, CP-super 1, and CP-super2 increased RIP3, but reduced cleaved Casp3 compared to TNFα. Using the ImageJ Gel Analysis method, the differences were quantitatively confirmed. These results suggest that the CP-spor-super1 and 2 may induce inflammatory response and cell necrosis in intestinal cells.

### 3.3. CP-Spor-Super1&2 Vaccines Reduced Severe NE-Induced Body Weight Gain Loss

**Figure 3:**
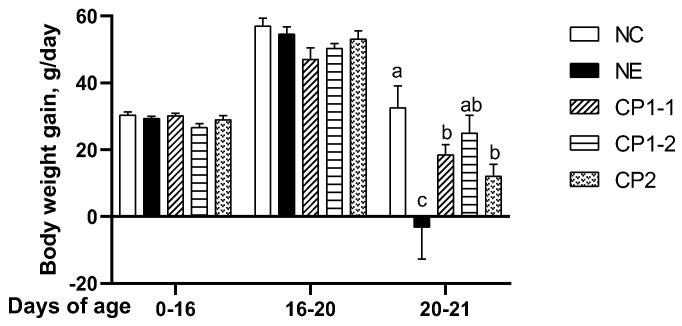
CP-spor-super vaccines protected birds against NE-induced body weight gain loss. Cohorts of 7–16 birds were fed basal diet and immunized with various CP-spor-super vaccines at d 0 and 10. To induce necrotic enteritis (NE), the birds were infected with 20,000 oocysts/bird *E. maxima* at 16 days of age and then infected with 10^9^ CFU/bird *C. perfringens* at 20 days of age. The birds were sacrificed at 21 days of age. The daily body weight gain during noninfected phase of d 0–16, *E. maxima* phase of d 16–20, and NE phase of d 20–21 are shown. All graphs depict mean ± SEM. Different letters of a, b, and c mean *p* < 0.05. Results are representative of two independent experiments.

Body weight gain is one of the most important parameters in the poultry industry, and acute NE is associated with dramatic body weight gain loss [10]. Because *C. perfringens* vegetative growth medium (toxoid) vaccine does not reduce NE in a *C. perfringens* infection alone model [46], we then only evaluated sporulation vaccines of CP-spor-super1 and 2. We evaluated two doses of CP-spor-super1 (CP1-1 (low dose), CP1-2 (high dose)) and one dose of CP-spor-super2 (CP2) as vaccines on influencing daily body weight gain (BWG) in NE birds. Notably, the vaccines did not have an apparent adverse effect on birds and the birds grew comparably between different groups during uninfected phase of d 0 to 16 and coccidiosis phase of d 16 to 20 (Figure 3). At the NE phase of d 20 to 21, NE-challenged birds showed the typical clinical NE signs of a decreased appetite, severe depression, watery to bloody (dark) diarrhea, closed eyes, and ruffled feathers, as reported before [14]. NE birds lost BWG compared to the uninfected negative control (NC) birds (−3.2 vs. 32.6 g/d). Remarkably, birds vaccinated with the three CP-spor-super vaccines of CP1-1, CP1-2, and CP2 were able to continue to BWG compared to NE birds’ BWG loss (18.5, 25, and 12.1 vs. −3.2 g/d, respectively). These results suggest that the three vaccines from CP1 and CP_2 protect birds against NE-induced BWG loss.

### 3.4. CP-Spor-Super1&2 Vaccines Attenuated Severe NE-Induced Inflammation in the Small Intestine

**Figure 4:**
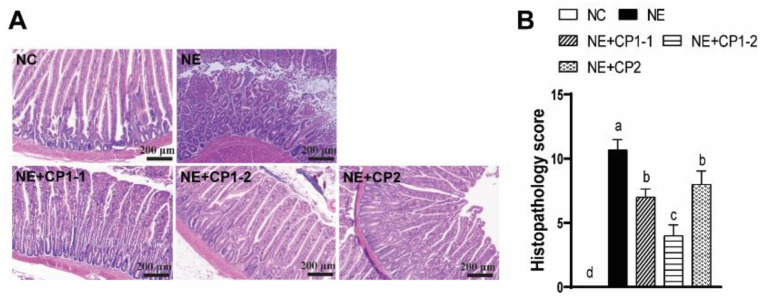
CP-spor-super vaccines protected birds against NE-induced intestinal histopathology. Birds were vaccinated and infected as in Figure 3 and bird numbers at d 21 were NC: 12; NE: 14; CP1-1: 16; CP1-2: 7; and CP2: 7. (**A**) H & E staining showing representative intestinal histology images. (**B**) Quantification of histological intestinal damage score. All graphs depict mean ± SEM. Different letters of a, b, c, and d mean *p* < 0.05. Results are representative of two independent experiments. Scale bar is 200 μm.

*E. maxima-* and *C. perfringens*-induced clinical NE is associated with inflammation in the small intestine [10]. We reasoned that the CP-spor-super vaccines would reduce NE-induced inflammation in the small intestine. The impact of the vaccines on the intestinal inflammation of chicken NE was investigated. During necropsy of the birds, the small intestine was gas distended and filled with foul-smelling and brown fluid. Upon dissection, the small intestine showed a thin and friable wall, covered with a thick layer of mucin, and spotted with macro-lesions across the segment. Upper intestinal tissue was collected as Swiss-Rolls, processed with H & E staining, and had histopathology analysis performed. Consistent with previous report [10], NE birds displayed severe intestinal inflammation as seen by the necrosis and fusion of villi and crypt, massive immune cell infiltration, and severe villus shortening (Figure 4A). Notably, the three vaccines of CP1-1, CP1-2, and CP2 attenuated NE-induced intestinal inflammation and histopathology score (7.2, 4.1, and 8.3 vs. 10.7, respectively) (Figure 4A,B). Interestingly, the CP1-2 vaccine reduced NE histopathology more compared to other vaccine regiments of CP1-1 and CP2.

### 3.5. CP-Spor-Super1 Vaccine Reduced C. perfringens Colonization and Invasion

**Figure 5:**
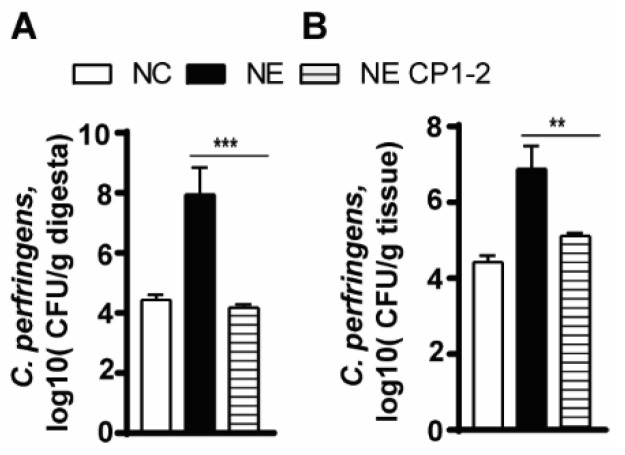
CP1-2 vaccine reduced *C. perfringens* colonization and invasion in the small intestine of NE birds. Bird experiment was conducted as in Figure 4. (**A**) Luminal *C. perfringens* colonization level in the intestinal content. (**B**) *C. perfringens* invasion level in the intestinal tissue. All graphs depict mean ± SEM. ***, *p* < 0.001; **, *p* < 0.01. Results are representative of two independent experiments.

Because *C. perfringens* colonization and invasion is associated with NE development, we then measured the bacterial infection in intestinal lumen and inside tissue of NE and CP1-2 birds using real time PCR. Notably, *C. perfringens* colonized at 3.5-log (Figure 5A) higher in the intestinal lumen of NE birds compared to those of NC birds. Notably, the CP1-2 vaccine reduced *C. perfringens* colonization by 47.4% in the small intestine digesta compared to NE birds. Consistently, the CP1-2 vaccine reduced *C. perfringens* invasion into the intestinal tissue (Figure 5B). These results suggest that CP-spor-super vaccines reduce *C. perfringens* colonization and invasion in the small intestine.

### 3.6. CP-Spor-Super1 Vaccine Reduced NE-Induced Immune Response

**Figure 6:**
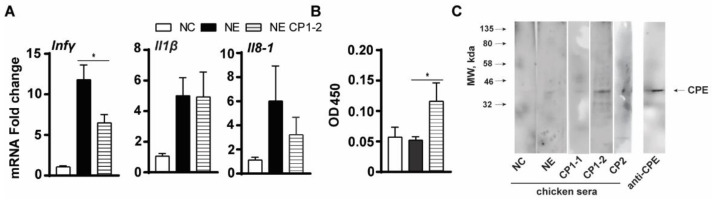
CP1-2 vaccine reduced NE-induced inflammatory response. Bird experiment was conducted as in Figure 4. (**A**) Proinflammatory gene expression in the small intestinal tissue. (**B**) ELISA result using antigen of CP-spor-super1 and primary antibody of individual serum from the birds. (**C**) WB result using antigen of CP-spor-super1 and primary antibody of group-pooled sera from the birds as well as anti-CPE primary antibody. All graphs depict mean ± SEM. *, *p* < 0.05.

Because of the reduction of intestinal inflammation and *C. perfringens* colonization and invasion by the vaccines, we then reasoned that the CP-spor-super1 vaccine would reduce the NE-induced pro-inflammatory response. We then measured host inflammatory mediator mRNA expression in the bird intestinal tissue using real-time PCR. The clinical NE induced higher accumulation of intestinal inflammatory mRNA mediators of *Ifnγ*, *Ilβ*, and *Il8-1* by 11.0, 4.7, and 6.1 folds, respectively, compared to NC birds (Figure 6A). Notably, the CP1-2 vaccine significantly reduced *Ifnγ* expression in the intestinal tissue by 45% compared to that in NE birds, while the vaccine did not significantly reduce other inflammatory cytokines. 

We then reasoned that the CP1-2 vaccine induced a strong humoral response of antibody production to reduce the inflammatory response. Serum samples from the individual birds were subjected to ELISA assay with antigen of CP-spor-super1. Notably, sera from CP1-2 birds induced significantly higher anti-CP-spor-super1 antibody compared to NE control birds (Figure 6B). To further identify which proteins in CP-spor-super1 induced the antibody production, we then performed a Western Blot assay using group-pooled serum samples. As showed in Figure 6C, the CP1-2 vaccine induced a strong band around 35 kda; the same molecular weight to anti-CPE band. Also noticeable were the additional molecular weight bands detected by the sera of CP1-2 birds. Interestingly, sera from NE birds showed a weak band around 35 kda, while no clear band was detected in noninfected NC birds.

## 4. Discussion

*C. perfringens*-induced enteritis are complex diseases with multiple factors including various intestinal segments and a variety of toxins associated with different susceptible hosts of humans and animals. Interestingly, the enteritis often shows combined manifestation of bacterial overgrowth [14,15], gas accumulation [16], collateral inflammatory self-damage response [10], and various toxin production [1]. Despite the different actions of the toxins, most of them are pore-forming toxins and induce cell death [1]. It is essential that *C. perfringens* and the toxins get access to internal tissues and anaerobic atmosphere through increased permeability of wound, inflammation, or others [47]. Hence, it is reasonable to reason that *C. perfringens*-induced enteritis among hosts of humans and animals shares certain common pathogenicity. Because the research on *C. perfringens*-induced human enteritis is limited by the lack of reliable animal models, enteritis studies from other animals such as chickens may render valuable insight on the mechanism of human enteritis or vice versa. Here, we found that at DNA level, CP1 and CP_2 were positive for *plc*, *cpb*, *cpe*, *iap*, *netB*, and *cola* genes, although mRNA or protein expression was not evaluated except for *cpe*. CP-spor-super1 and 2 induced inflammatory response and cell necrosis in macrophage Raw 264.7. Importantly, birds immunized with the CP-spor-super1 and 2 were resistant to *C. perfringens*-induced severe clinical NE on histopathology and BWG loss. The CP1-2 vaccine reduced NE-induced proinflammatory gene expression and *C. perfringens* colonization and invasion. Together, this study showed that CP-spor-super vaccines reduced NE histopathology and productivity loss, suggesting that *C. perfringens* sporulation plays an essential role on mediating the enteritis.

This study demonstrated that vaccines using *C. perfringens* sporulation proteins reduced chicken NE. *C. perfringens* is a multiple toxin-producing bacterium, including CPA, CPB, ETX, ITX, CPE, NetB, and others [48]. One of effective approaches to assess the role of the toxins or other virulent factors on *C. perfringens*-induced enteritis is to immunize the animals with the suspected molecules to invoke protective immunity. Because of the toxin knowledge and the affordable cost of vaccines, various vaccines have been investigated targeting the toxins or other *C. perfringens* molecules in chicken NE. There are generally two types of NE infection models: *C. perfringens* infection alone with various dietary manipulation, or *Eimeria* and *C. perfringens* co-infection. In *C. perfringens* infection alone studies, *C. perfringens*-induced mild lesions were reduced in birds vaccinated with recombinant proteins of PlcC (CPA), glyceraldehyde-3-phosphate dehydrogenase, pyruvate:ferredoxin oxidoreductase, fructose 1,6-biphosphate aldolase (Fba), large cytotoxin (TpeL), endo-beta-N-acetylglucosaminidase, and phosphoglyceromutase [49,50]. *C. perfringens*-induced lesions were partially reduced in birds vaccinated with rNetB, but not bacterins [46]. The regulated delayed lysis *Salmonella* vaccine expressing PlcC and GST-NetB elicited intestinal IgA, IgY, and IgM antibodies and protected broilers against *C. perfringens* challenge [51]. The *Salmonella* vaccine expressing Fba alone or NetB, PlcC, and Fba protected broilers against *C. perfringens* challenge [52]. However, NE in commercial poultry production is often associated with coccidiosis and has more severe and complex disease manifestation compared to the *C. perfringens* infection alone model in the above-mentioned vaccine studies. Hence, the role of *C. perfringens* toxins or other molecules on NE development remains largely inconclusive in the poultry industry, which is evident from the fact that there are no known effective toxins or enzyme vaccines to control NE in the field [53]. It remains much needed to develop effective vaccines against NE in poultry farms. Indeed, the most practical methods to prevent NE in poultry farms is to use the coccidia vaccine, however concerns often arise with the occasional outbreak of coccidiosis from live coccidia vaccines [4]. Our report may encourage additional studies on *C. perfringens* pathogenesis and vaccine development using the co-infection model, which is relevant to poultry farm settings.

*C. perfringens* is a sporulation bacterium, and sporulation-associated CPE forms cation-permeating pore to cause the influx of calcium and cell death [54]. CPE is the main toxin implicated in the enteritis in humans [48], and it is interesting that the role of sporulation on chicken NE has not been reported. In this study, CP-spor-super1 and 2 induced cell necrosis and inflammatory response, suggesting the presence of bioactive toxin(s) in the CP-spor-super. Interestingly, the CP-spor-super1 vaccine reduced NE histopathology and BWG loss better compared to the CP-spor-super2 vaccine, although the expression level of CPE was less in CP-spor-super1 compared to CP-spor-super2. The result led to the reasoning that *C. perfringens* sporulation proteins other than CPE or toxin(s) might also play important role on generating protective immunity against chicken NE. The notion of non-toxin proteins mediating NE were implicated before. For example, *C. perfringens* enzymes of Fba, phosphoglyceromutase, endo-beta-N-acetylglucosaminidase, and phosphoglyceromutase were used to immunize birds and protect chickens against NE [49,50]. It remains largely unknown what the role of the enzymes on *C. perfringens*-induced enteritis. It is well documented that immunization-generated antibody is responsible for reducing bacterial infection [55]. The increased ELISA reading in CP1-2 group sera suggested the success of anti-CP-spor-super1 Ab production. Interestingly, Western Blot showed multiple bands detected by CP1-2 group sera, while the strongest band was the same size of CPE. The Western Blot results did not exclude the possibility of the presence of other toxins, such as NetB, in the supernatant. However, from the literatures, only CPE is expressed during sporulation [54]. It is inconclusive how important it is to generate NE protective immunity from other toxins/proteins naturally expressed and secreted during in vitro vegetative culture. This is indicated by the report that the birds were not protected by vaccines with *netB* positive *C. perfringens* supernatant alone (toxoid) or bacterin (50/50% bacteria and toxoid) using the high protein diet, only *C. perfringens* challenge NE model [46]. Only vaccines combined with recombinant NetB, toxoid, and bacterin protected the birds from the NE. The results suggested that in vitro naturally expressed (not overexpression) and secreted NetB as a vaccine fails to produce the protective immunity, and additional antigens/toxins and overexpressed NetB are needed to generate the protection in the NE challenge model. We have performed proteomics of the supernatants, but the results (not shown) were very complicated, and we are working on analyzing them. We are also working on finding the immunogenic proteins in the CP-spor-super using Phage Display and sera from the immunized chickens. The identification of sporulation proteins may reveal the role of important *C. perfringens* proteins on mediating the enteritis in chickens, other animals, and humans.

At the cellular level, NE causes severe small intestinal inflammation, demonstrating as massive immune cells migrating into the lamina propria, villus demolishment, and crypt hyperplasia [56,57]. Intestinal inflammation is essential for clearing invaded microbes and to resolve inflammation, however overzealous inflammation often induces more bacterial invasion and collateral tissue destruction and inflammation [58]. One consequence of over inflammation in enteritis is the destruction of epithelial and immune cells in the lamina propria [59]. In addition, infectious bacteria often hijack the inflammatory response to gain a survival and invasion advantage. Coccidia infection induces strong immune response and intestinal inflammation in chickens [60,61]. The “hyper-inflammation” by coccidiosis and *C. perfringens* infection in NE birds might lead to the severe intestinal inflammation demonstrating as massive immune cell infiltration, elevated inflammatory cytokine expression, and epithelial cell hyperplasia and death in this study and as described before [10]. Consistent with previous report [14], NE in this study induced strong inflammatory gene expression of *Infγ*, *Il1β*, and *Il8-1* as well as cell death. Interestingly, the CP1-2 vaccine attenuated *Infγ*, *C. perfringens* invasion and intestinal cell death without significant reduction of *Il1β* and *Il8-1*. Besides the humoral adaptive response of producing antigen-specific Ab, immunization also induces the cellular adaptive response of activating CD8 T cells [55]. It would be helpful for further research to investigate how the vaccine mediates host innate and adaptive response and reduces inflammation and cell death in relation with impacting *C. perfringens* pathogenesis.

## 5. Conclusions

Altogether, these data reveal that vaccines using *C. perfringens* sporulation proteins reduce NE in chickens, through reducing the NE-induced host inflammatory response and cell death as well as the pathogen colonization and invasion. These findings highlight the importance of elucidating the molecular relationship between the infectious pathogen and host response. These discoveries could be applied to control *C. perfringens*-induced enteritis and other intestinal diseases targeting pathogen virulence factors and host inflammatory response.

## Data Availability

Data are presented in this paper.

## Supplementary Materials

The following supporting information can be downloaded at: https://www.mdpi.com/article/10.3390/microorganisms10061110/s1, Supplementary Table S1: Primers used in this study; Supplementary Figure S1. DCA reduced *C. perfringens* invasion and sporulation in the small intestine of NE birds.
