# Supplementary material for: Vaccines Using Clostridium perfringens Sporulation Proteins Reduce Necrotic Enteritis in Chickens
Source: Microorganisms. 2022 May 27;10(6):1110. doi: 10.3390/microorganisms10061110 (PMC9228780; doi:10.3390/microorganisms10061110)
Supplement: Supplementary file 1 [file microorganisms-10-01110-s001.zip › microorganisms-1699145-SI.pdf]

Supplementary Materials

Materials and Methods

Chicken experiment

Animal experiments were conducted in accordance with the Animal Research: Reporting of *In Vivo* Experiments (https://www.nc3rs.org.uk/arrive-guidelines) and were approved by the Institutional Animal Care and Use Committee of the University of Arkansas. Cohorts of thirteen one-day-old broiler chicks per group were obtained from Cobb-Vantress (Siloam Springs, AR). Chicks were neck-tagged and randomly allocated to floor pens with new pine shavings as litter in an environmentally controlled isolated room. The birds were raised on floor pens with their respective diet and water *ad libitum*, and temperature was maintained at 34°C for the first 5 days of age and was then gradually reduced until a temperature of 23°C was achieved at day 26 days of age. The birds were fed a corn-soybean meal-based starter diet during 0-10 days of age and a grower diet during 11-26 days of age. Treatment diets were supplemented with 1.5 g/kg DCA (Alfa Aesar). No antibiotics, coccidiostats or enzymes were added to the feed. An aliquot of frozen *C. perfringens* was grown in TSB plus sodium thioglycolate overnight for the NE challenge study and was serially diluted and plated on tryptic soy agar plus sodium thioglycolate for enumerating CFU. Birds were infected with 20,000 sporulated oocytes/bird M6 *E. maxima* at 18 days of age and 10^9 CFU/bird *C. perfringens* at 23 and 24 days of age. The ileal tissue was Swiss-rolled for histopathology analysis.

Fluorescence in situ hybridization (FISH)

FISH was performed to visualize *C. perfringens* intestinal colonization and invasion using histology slides. The FISH with probe of Cp85aa18: 5′-/Cy3/TGGTTGAATGATGATGCC-3′ was performed similarly to previous report [10] with modification of bacterial DNA exposure by boiling. Briefly, deparaffinized, formalin-fixed 5 µm thick sections were immersed in 10 mM sodium citrate buffer pH 6.0, boiled and then maintained at a sub-boiling temperature for 10 minutes. After cooled and washed, the slides were incubated with the FISH probe at a final concentration of 5 ng/µl overnight at 46 °C in humid 50 ml tubes. The slides were washed for 20 min at 48 °C in washing buffer and once in distilled water for 10 seconds. The slides were counterstained with DAPI for 2 min and dried at room temperature and mounted with 50% glycerol. The FISH were visualized using a Nikon TS2 fluorescent microscopy and representative images of each group were presented in the Results section.

Supplementary Table S1: Primers used in this study

Gene	Forward Primer Sequence (5′-3′)	Reverse Primer Sequence (5′-3′)	Trials
Cp_*plc*	TGACACAGGGGAATCACAAA	CGCTATCAACGGCAGTAACA	PCR
Cp_*cpe*	CAACTGCTGGTCCAAATGAA	GCATCTTTCGCCAGTTTCAA	PCR
Cp_*cpb*	AAGTTTGTACATGGGATGACGA	CCCCACATCCAATGGTCTAC	PCR
Cp_*etx*	AGATGGAGAACCATCAATGAA	GCTTGTATCGAAGTTCCCACA	PCR
Cp_*iap*	CTCTGACCACGCAAGCTTTA	TTCGCTCCATACCACAGAGA	PCR
Cp_*ibp*	TGAGAAAGCTTCCGGTTCTT	GCCCTGATCAGTTGAAGCAT	PCR
Cp_*netB*	GGAAAAATGAAATGGCCTGA	GCACCAGCAGTTTTTCCTTC	PCR
Cp_*colA*	TAGGAACAAAGGCGCAAGAT	GAATACTGCATTCCCCTTGC	PCR
Cp_16S	CAACTTGGGTGCTGCATTCC	GCCTCAGCGTCAGTTACAGT	PCR
chicken *Il8-1*	CCTCCTGCCTCCTACATTCA	ATCTCCAGCTCCTTTCACGA	Real-time PCR

Supplementary Figure S1

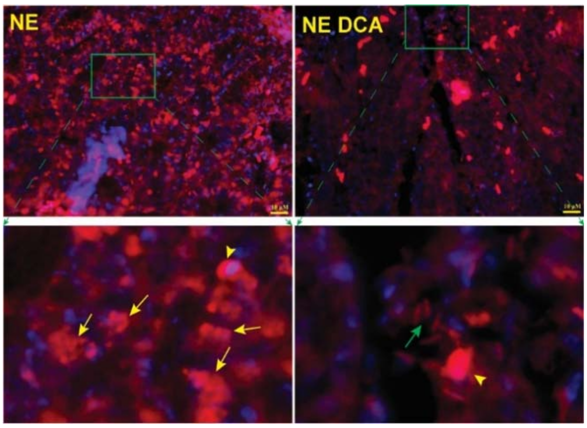

Supplementary Figure S1. DCA reduced *C. perfringens* invasion and sporulation in the small intestine of NE birds. Cohorts of one-day-old chicks were fed 0 (basal diet) or 1.5 g/kg DCA diets. To induce necrotic enteritis (NE), the birds were infected with 20,000 oocysts/bird *E. maxima* at 18 days of age and then infected with 10^9 CFU/bird *C. perfringens* at 23 and 24 days of age. The birds were sacrificed on 26 days of age and the data were reported before [10]. Yellow arrows - spores, green arrow - vegetative cells, and yellow arrow heads – red blood cells. Scale bar is 10 μm.
